# Supramolecular Hydrogels for Protein Delivery in Tissue Engineering

**DOI:** 10.3390/molecules26040873

**Published:** 2021-02-07

**Authors:** Yaqi Lyu, Helena S. Azevedo

**Affiliations:** School of Engineering and Materials Science, Institute of Bioengineering, Queen Mary University of London, Mile End Road, London E1 4NS, UK; y.lyu@qmul.ac.uk

**Keywords:** supramolecular interactions, hydrogels, injectable, stimuli-responsive, controlled release, proteins drugs, tissue engineering

## Abstract

Therapeutic proteins, such as growth factors (GFs), have been used in tissue engineering (TE) approaches for their ability to provide signals to cells and orchestrate the formation of functional tissue. However, to be effective and minimize off-target effects, GFs should be delivered at the target site with temporal control. In addition, protein drugs are typically sensitive water soluble macromolecules with delicate structure. As such, hydrogels, containing large amounts of water, provide a compatible environment for the direct incorporation of proteins within the hydrogel network, while their release rate can be tuned by engineering the network chemistry and density. Being formed by transient crosslinks, afforded by non-covalent interactions, supramolecular hydrogels offer important advantages for protein delivery applications. This review describes various types of supramolecular hydrogels using a repertoire of diverse building blocks, their use for protein delivery and their further application in TE contexts. By reviewing the recent literature on this topic, the merits of supramolecular hydrogels are highlighted as well as their limitations, with high expectations for new advances they will provide for TE in the near future.

## 1. Introduction

Tissue engineering (TE) has emerged as a result of bioengineering breakthroughs in the early 1990s [1] aiming to repair malfunction tissues in the body caused by genetic mutations, congenital abnormalities, aging, disease or injury. TE approaches are focused on the combined use of (1) cells, as building workers to repair and produce new tissue, (2) scaffolds, to support and guide cells and (3) biomolecules, which are able to regulate the fate of cells. Typically, those biomolecules are bioactive proteins, like growth factors (GFs), that regulate cell proliferation, differentiation, migration and other cell behaviors during tissue development [2,3]. The use of bioactive proteins has been widely exploited in TE, not only because of their direct effects on cells but also due to the rapid development of biotechnology, in particular the production of recombinant proteins. Direct administration of bioactive proteins in the body is known to be poorly controlled and can lead to undesired effects. Besides, the half-life of protein drugs in serum is very short, often within hours, requiring repeated dosing to maintain sufficient concentrations to produce therapeutic effects. To improve the availability of bioactive proteins, elegant delivery systems have been designed for their controlled and sustained release.

**Figure 1:**
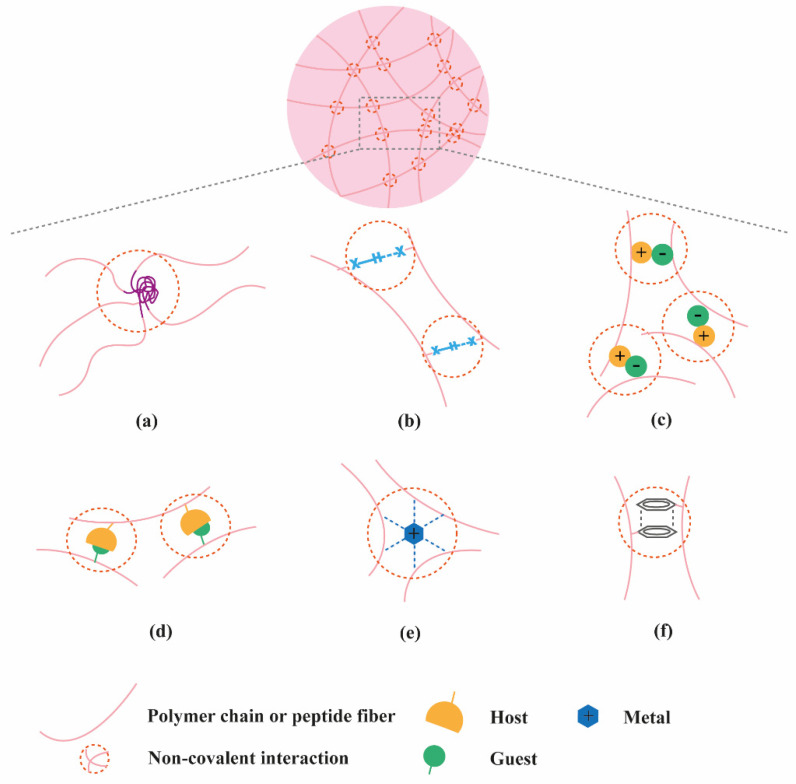
Application of supramolecular chemistry to create physically crosslinked hydrogels. (**a**) hydrophobic effects; (**b**) hydrogen bonding; (**c**) electrostatic interactions; (**d**) host-guest interactions; (**e**) metal ligand interactions; (**f**) π-π stacking.

Hydrogels have become popular materials in biomedical applications due to their generally accepted biocompatibility and wide range of properties, from soft to stiff, to stimuli-responsive and cell-instructive. Hydrogels own a three-dimensional structure rich in water and held by a network of hydrophilic polymers. This architecture resembles the native extracellular matrix (ECM) in tissues. As such, hydrogels have been also highly considered for TE applications where they can hold cells [4] and provide mechanical support [5]. In addition, the properties of hydrogels offer various possibilities for the controlled delivery of proteins: (1) The massive water content enables the easy encapsulation of water-soluble molecules such as proteins; (2) The cross-linked network and composition of the hydrogels can be tailored, allowing control over the mesh size and thus the possibility to govern the release of entrapped proteins, based on their size and affinity to the hydrogel components; (3) The hydrated network provides protection to entrapped proteins against proteolytic degradation and prolongs their bioactivity. Based on the crosslinking method, hydrogels can be classified into two main types: chemically (through covalent bonds) and physically (or supramolecular) crosslinked hydrogels. Supramolecular hydrogels are formed via non covalent interactions such as hydrogen bonding, hydrophobic effects, host–guest recognitions, electrostatic interactions, metal-ligand interactions, π-π interactions and van der Waals forces (Figure 1).

**Figure 2:**
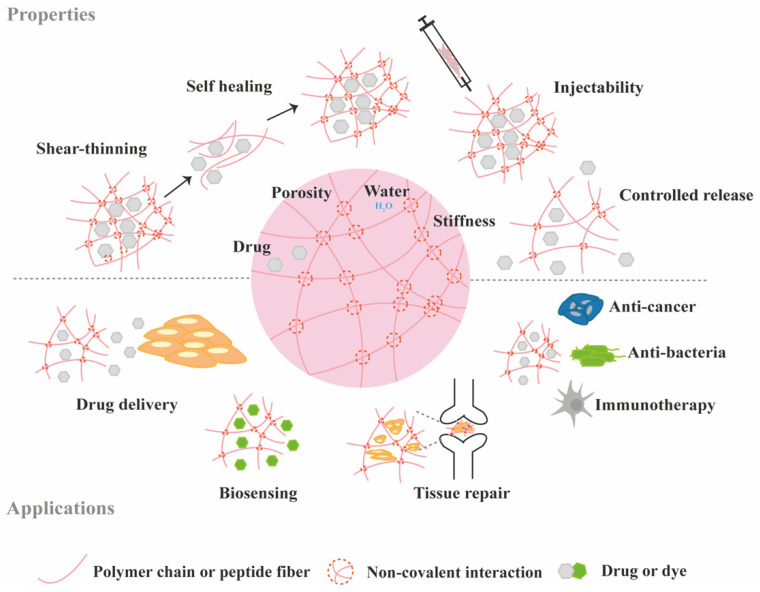
Schematic highlighting the properties and medical applications of supramolecular hydrogels.

Generally, supramolecular hydrogels are formed under mild environmental conditions, which enables the direct addition of sensitive molecules, such as proteins, during hydrogel formation. The dynamic nature of non-covalent interactions in supramolecular hydrogels, allows their minimally invasive delivery by injection. In addition, the dense crosslinked network can prevent diffusion of proteolytic enzymes and is thus believed to protect bioactive therapeutics from premature degradation [6]. The reversible nature of noncovalent crosslinking also provides repeated release on demand, as they are able to disassemble and reassemble based on environmental stimuli [7]. Figure 2 highlights the properties and medical applications of supramolecular hydrogels. Compared to supramolecular hydrogels, most hydrogels crosslinked by non-dynamic covalent bonds are unable to undergo crosslinking again after breaking and recover the original properties and function. Covalent bonding will decrease the flexibility of the hydrogels, making them difficult to integrate with the dynamic environment of native tissues [8,9]. Hence, the unique properties of dynamic and reversible noncovalent interactions make supramolecular hydrogels an ideal protein delivery system for TE applications. Table 1 provides a general comparison between hydrogels crosslinked by permanent covalent bonds and by supramolecular forces regarding their properties with relevance for protein delivery in TE applications.

This mini review will focus on the use of supramolecular hydrogels for the delivery of proteins in TE. We start by describing the various types of supramolecular hydrogels and how they have been designed, even if their applications in TE have not been demonstrated. The aim is to provide an overview on the repertoire of supramolecular hydrogels reported in the literature and with potential utility in TE. The possibility to control the kinetics of protein release from supramolecular hydrogels is discussed through examples utilizing model proteins. Next, the application of supramolecular hydrogels for the controlled release of bioactive proteins in the context of TE is described including angiogenesis, bone and cartilage regeneration and wound healing. Finally, supramolecular hydrogel/protein formulations that have reached clinical trials are shortly discussed to provide a better understanding of the clinical application prospects of supramolecular hydrogels.

## 2. Classification of Supramolecular Hydrogels Based on Their Composition

### 2.1. Polymer-Based Hydrogels

Polymer-based supramolecular hydrogels can be from natural or synthetic origin. The most popular advantages of natural polymers are their biocompatibility and biodegradation which are key in TE applications. Polysaccharides are a series of hydrophilic natural polymers including dextran, chitosan, hyaluronic acid, alginate, cellulose among others.

Dextran (Dex) is a water-soluble polysaccharide consisting of α-1,6-linked D-glucopyranoses and the hydroxyl groups in dextran can be conjugated with functional groups for the formation of a crosslinked structure. Chen et al. [10] conjugated dextran with 2-naphthylacetic (2-NAA) through ester bond and hyaluronic acid with β-cyclodextrin (β-CD) to form supramolecular hydrogel (HA-Dex) by host-guest interaction between β-CD 2-NAA. Cell culture experiments demonstrated that NIH-3T3 fibroblasts could adapt to the microenvironment formed by HA-Dex hydrogels making HA-Dex a potential material as cell scaffold. Dextran can also be modified into carboxymethyl dextran (CMDH) and subsequently to aminodextran (AD) which can then be utilized as additives with a derived C2-phenylalanine gelator (LPF) [11]. LPF interacted with CMDH and AD via hydrogen bonding and π-π stacking respectively, resulting in enhanced mechanical stability of the hydrogel. 

Chitosan is linear polysaccharide with cationic nature, composed of randomly distributed β-(1-4)-linked d-glucosamine and *N*-acetyl-D-glucosamine units [12]. Free amino and hydroxyl groups of chitosan can be easily modified to incorporate functional groups amenable for supramolecular interactions. An injectable supramolecular carboxymethyl chitosan-zinc (CMCh-Zn) hydrogel was prepared for antibacterial applications [13]. The coordination of empty orbitals of Zn^2+^ with lone pair of electrons of NH_2_, OH and COO^−^ groups of CMCh leads to the rapid formation of CMCh-Zn complex after simply mixing a solution of modified chitosan and Zn(NO_3_)_2_·6H_2_O salt together within the pH range of 5.3–7.0. These CMCh-Zn hydrogels could be used for bone TE applications as Zn is an essential element in bone homeostasis and has been used as a therapeutic agent in bone regeneration [14,15]. However, the use of metal ions should be carefully considered as they can be toxic if exceeding tolerable concentrations. 

**Figure 3:**
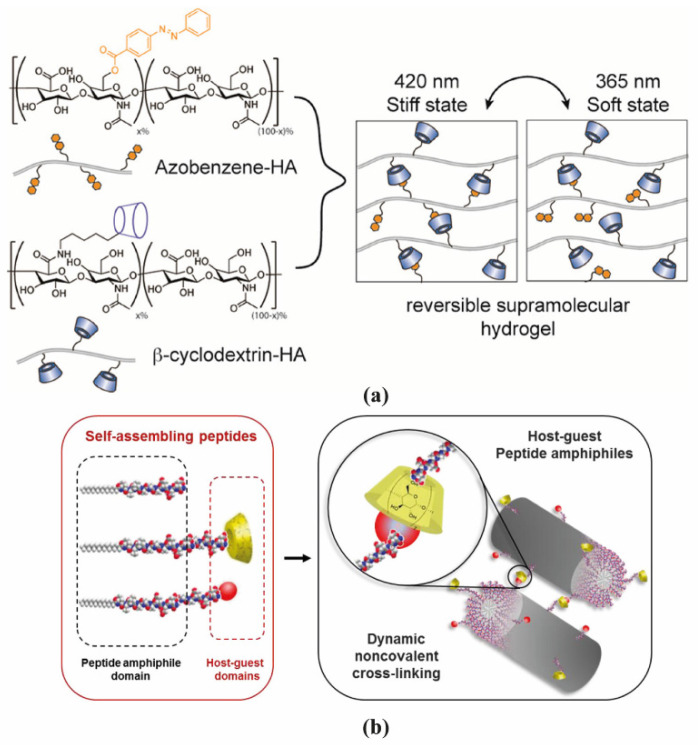
Representative supramolecular hydrogels based on host-guest interaction. (**a**) Polymer-based host-guest hydrogel between HA-β-CD and HA-Azo. With UV-irradiation at 365 nm, azobenzene will transform from its trans-state to cis-state, leading to gel disassembly. The gelling process is reversible with irradiation of visible light (400–500 nm). Adapted with permission from Ref. [17] Copyright © (2018), American Chemical Society. (**b**) Peptide-based host-guest hydrogel between PA-β-CD and PA-Ad. Incorporating β-CD and Ad interaction in the PA nanofiber improves the stability of PA-based hydrogel. Adapted with permission from [21] Copyright © (2019), American Chemical Society.

Hyaluronic acid (HA) is a linear polysaccharide composed of repeating disaccharide units of d-glucuronic acid and *N*-acetylglucosamine. Burdick’s group developed an HA-based supramolecular hydrogel based on β-CD-modified HA (HA-β-CD) and adamantane-modified HA (HA-Ad) through host-guest interactions between CD and Ad [16]. This HA-based hydrogel is shear-thinning and could rapidly recover its gel form at injection site, indicating its great potential for non-invasive delivery. The hydrogel was upgraded by modification of HA with azobenzene (Azo), a light sensitive molecule, instead of Ad [17]. The host-guest interaction between CD and Azo could be modulated by light with different wavelength, as shown in Figure 3a, which was able to tune the release of entrapped protein. HA was also modified with a hydrophobic molecule to obtain amphiphilic HA. Cholesterol was conjugated to HA as building block, which could self-assemble into an injectable nanohydrogel [18,19]. The self-assembly ability of cholesterol-grafted-HA (HA-CH) appears owing to the hydrophobic interactions between the cholesterol cores and the hydrophilic interactions between HA shells. In addition, due to the presence of hyaluronidase in tissues, HA-based hydrogels are able to be enzymatically degraded to achieve a complete release of the entrapped cargos [20].

Synthetic polymers can be designed and synthesized with specific functional groups to obtain hydrogels with on-demand physical properties. The synthetic polymer poly(ethylene glycol) (PEG) has been widely investigated in TE applications due to its easy functionalization. A great number of supramolecular hydrogels were prepared via PEG-based polymers. β-CD and cholesterol were conjugated to star-shaped PEGs and supramolecular hydrogels were shown to self-assemble via hydrophobic and van der Waals interactions between β-CD and cholesterol [22]. The ability of the star-shaped PEG based hydrogels as protein delivery vehicles and the release profile of model proteins from these hydrogels were then investigated. The cross-link density and swelling stresses played prominent roles in controlling the release kinetic. On the one hand, after absorption of water, the increased swelling stresses accelerated the dissociation of β-CD/cholesterol complexes. On the other hand, the flexible polymer was able to relieve some swelling stresses to slow down the dissociation of the complexes. Hence, a nearly zero-order release of the entrapped proteins was achieved with the balance between the two mechanisms [23].

**Figure 4:**
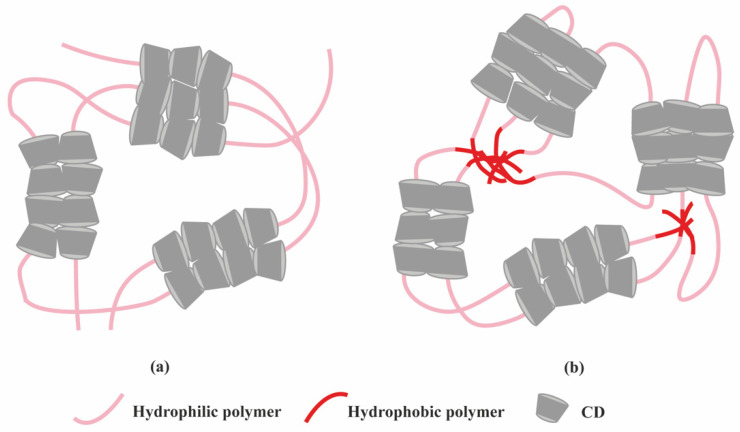
Scheme showing the formation of supramolecular hydrogels by polymer–CD inclusion complexes. (**a**) Threading of CD onto hydrophilic polymers; (**b**) Threading of CD onto amphiphilic polymers.

Another class of supramolecular hydrogels receiving great attention in drug delivery applications are based on polymer–CD inclusion complexes [24]. It has been shown that hydrophilic polymers such as PEG could penetrate the inner cavity of α-CD forming an inclusion complex with a necklace-like structure [25]. These polymer–CD inclusion complexes can self-assemble, via aggregation of the inclusion domains, and lead to the formation of a physically crosslinked hydrogel (Figure 4a). In these systems, drug incorporation can be achieved in aqueous environment during the gelation process making it attractive for protein delivery. Using poly(caprolactone)-poly(ethylene glycol)-poly(caprolactone) (PCL-PEG-PCL) triblock copolymer and γ-cyclodextrin (γ-CD), an injectable supramolecular hydrogel was developed for insulin delivery [26]. The inclusion complexes were formed directly by the PEG segment in PCL-PEG-PCL backbone and γ-CD, not requiring conjugation with additional guest molecules. The ratio between PEG and PCL determined the formation of the hydrogel. A certain amount of hydrophilic PEG could keep a balance between hydrophobic (PCL) and hydrophilic (PEG) segments of the copolymer and increase the chance of γ-CD to thread onto the PEG blocks, since hydrophobic interaction between PCL segments acts as a barrier against γ-CD threading. PEG blocks were covered by γ-CD when inclusion complexes were formed, thus enhancing the opportunity of hydrophobic interactions via PCL segments, leading to the rapid gel formation (Figure 4b).

Stimuli-responsive hydrogels have been also exploited for the delivery of therapeutic proteins. These smart hydrogels undergo phase transition in response to external changes in the environment such as temperature, pH, light, magnetic [27] and electrical fields [28]. The external stimuli can be accurately regulated to achieve precise control over protein release. 

Synthetic polymers with lower critical solution temperature (LCST) are suitable for design of injectable thermo-sensitive hydrogels, including poly(ethylene oxide)-*b*-poly(propylene oxide)-*b*-poly(ethylene oxide) (PEO-PPO-PEO) [29], poly(vinyl ether)s (PVEs) [30] and a series of *N*-substituted acrylamide polymers such as poly(*N*-isopropylacrylamide) (PNIPAm), poly(*N*,*N*-diethylacrylamide) (PDEAm), poly(*N*-vinyl-*n*-butyramide) (PNVBAm), among others. For example, PNIPAm has an LCST at 32 °C, which is higher than room temperature, but lower than body temperature, meaning PNIPAm can easily reach sol-gel transition after injection in the body. Modification of PNIPAm with acryloyl-β-cyclodextrin (Aβ-CD) was found to decrease the LCST to 28–30 °C with different conjugation rates, indicating LCST can be slightly affected by modification of the thermo-sensitive polymer [31]. β-CD modified PNIPAm and adamantyl-terminated poly(ethylene glycol) (Ad-PEG) were synthesized to form dual supramolecular assemblies using the host-guest interaction between β-CD and adamantyl group, together with the formation of polypseudorotaxane between α-cyclodextrin (α-CD) and PEG chains with additional α-CD added into the system [32]. When the temperature increased from 25 to 37 °C, the hydrophobic interactions of PNIPAm segments would become the dominant force, making hydrogels stronger. Conversely, a thermo-sensitive response could also contribute to a controlled release profile when the hydrogels undergo gel-sol transition. Another thermo-sensitive hydrogel using host–guest interaction was prepared using an amphiphilic copolymer pyrene-poly(caprolactone)-*b*-poly(oligo(ethylene glycol) methacrylate) (Py-PCL-*b*-POEGMA) and α-CD at room temperature [33]. α-CD acted as the host molecule while POEGMA acted as guest molecule. BSA was loaded in this thermo-sensitive hydrogel and a faster release at 37 °C was achieved compared to 25 °C. The temperature-dependent behavior of the release results from the dissociation of α-CD when the temperature is increased. Therefore, the hydrogels suffered partly from structural damage at higher temperature and faster release was observed.

Similar to temperature responsiveness, pH changes were also utilized to trigger phase transitions of supramolecular polymer hydrogels taking advantage of pH differences in different parts of body. Hydrogen bonds and electrostatic interactions are pH-sensitive. Changes in pH affect the protonation/deprotonation of acidic/basic groups on polymers. For example, a synthetic, catheter-injectable supramolecular hydrogel was fabricated by ureido-pyrimidinone (UPy) units and poly(ethylene glycol) (PEG) chains via hydrogen bonding. These UPy-modified PEG hydrogels formed fibers in aqueous solution and were able to undergo gel-sol transition at basic pH, and reversibly turn back to gel state at neutral pH. The fast pH switchability of UPy-modified PEG hydrogels was likely caused by the breaking down of crosslinks between the fibers that form the transient network instead of complete disassembly of the fibers [34]. A pH-responsive supramolecular hydrogel was prepared based on poly(acryloyl 6-aminocaproic acid) (PA6ACA) and poly(methacrylic acid-co-ethyl acrylate) (EUDRAGIT L 100-55). In the acidic environment, intermolecular hydrogen bonds between carboxyl groups and amide units on PA6ACA and EUDRAGIT L100-55 drove the assembly of the supramolecular network. At neutral aqueous environments, the hydrogen bonds were eliminated due to the deprotonation of carboxylic acid groups, leading to the gel-sol transition [35].

Another strategy to build stimuli triggered supramolecular hydrogels is to modify the polymer with functional groups that are sensitive to light. Light stimulus is different from temperature or pH stimuli since it can be precisely controlled outside the body. One example that has been widely exploited is the use of *cis*-*trans* isomerization to generate light-responsive supramolecular hydrogels. The phase transition of this kind of hydrogel is usually based on host-guest interaction. As previously mentioned, HA was functionalized with CD and Azo to obtain a light-responsive supramolecular hydrogel. Tamesue et al. [36] reported a similar light responsive supramolecular hydrogel prepared by CD-conjugated curdlan (CD-CUR) and Azo-modified poly(acrylic acid)(pAC_12_Azo). The supramolecular hydrogels were fabricated under visible light and underwent gel-sol transition upon irradiation at 365 nm, which resulted in the transition from trans isomer to cis isomer of Azo group. Recently, the CD- and Azo-based light-responsive supramolecular hydrogel was modified by using upconverting nanoparticles [37], in order to induce the phase transition of hydrogel under NIR irradiation, which has a deeper tissue penetration and is safer for in vivo applications than UV light. Derivatized Azo (modified with an alkoxypropanol (azopropOH) side group) was conjugated to poly(acrylic acid) which interacted with deoxycholate-β-CD to form host-guest complex. LiYF_4_:Tm^3+^/Yb^3+^ upconverting nanoparticles (LnUCNPs) were immobilized in the hydrogel matrix to provide upconversion effect in UV-region. Due to the high viscosity environment of the supramolecular hydrogel, the Brownian motion of LnUCNPs was limited which increased the chance for energy transfer to achieve phase transition. The unmodified supramolecular hydrogel went through the gel-sol transition under 365 nm irradiation in 25 min. When incorporated with LnUCNPs, the resulting light responsive supramolecular hydrogel was found to have a gel-sol transition time of 60 min under 980 nm excitation. Although a long time was needed, the successful NIR response indicates its potential to act as photo-responsive delivery system for applications in deep tissue. Another strategy to achieve light sensitivity is to use photo-cleavable moieties, but this strategy is often used to prepare chemically crosslinked hydrogels and will not be discussed in this review.

### 2.2. Peptide-Based Hydrogels

The self-assembly ability of peptides has attracted great attention to develop supramolecular hydrogels. Peptides have been engineered with various molecular motifs (α-helix, β-sheet, amphiphilic, collagen- and elastin-like) to self-assemble into filaments, which at certain concentration can entangle and form a nanofibrous hydrogel network.

A designed supramolecular hydrogel formed by hydrogelating self-assembling fibers (hSAFs) was reported by Mehrban et al. [38]. Two peptides gelled together and formed coiled-coil α-helical fibrous nanostructures. Subsequently, the cell adhesion motif RGDS was attached to the peptide fibers containing azide functionality via a click reaction with alkyne-RGDS for integrin binding. Images from scanning electron microscopy (SEM) showed interconnected fibers and porous structure in both hydrogels with or without RGDS, indicating the stability of coiled-coil fibrous structures. Similar approach could be used to attach protein molecules onto hSAFs.

Peptides designed to self-assemble with β-sheet structure normally requires repeat sequences of ionic hydrophilic and hydrophobic amino acids, such as AEAEAKAKAEAEAKAK (AEAK16-II) [39]. The peptide sequence forms β-sheet structure with hydrophobic face on one side and hydrophilic face on the other side, with the hydrophobic in the fiber core contributing to the stability of the structure. The electrostatic interactions and hydrogen bond between β-sheet layers result in the formation of fibrils. Both small molecules and biomacromolecules could be entrapped between these fibrils for sustained release by modulating the fiber density. A two-layered nanofiber hydrogel was formed by Ac-(RADA)_4_-NH_2_ and Ac-(KLDL)_3_-NH_2_ self-assembling peptides with Ac-(RADA)_4_-NH_2_ in the core layer and Ac-(KLDL)_3_-NH_2_ in the shell layer. The mechanical properties, as well as the hydrogel network density, could be altered by adjusting the density of Ac-(KLDL)_3_-NH_2_. In addition, the initial burst release of protein from this two-layer hydrogel was decreased compared to the single peptide formed hydrogel, which resulted from the higher nanofiber density provided by the additional layer [40]. The morphology of a self-assembled β-sheet pentapeptide hydrogels could be tuned by altering the charge distribution of the peptide sequence [41]. The pentapeptide contains three aliphatic isoleucine (I) residues, with potential to form β-sheets, and two aspartic acid (D) residues to improve solubility (DIIID-NH_2_, DDIII-NH_2_ and IDIDI-NH_2_). These three pentapeptide sequences can form robust hydrogels with gelation induced through changes in pH. Morphology examination by cryo-focused ion beam SEM showed IDIDI-NH_2_ hydrogels were formed by high aspect-ratio nanofibers while the DDIII-NH_2_ and DIIID-NH_2_ hydrogels were made of more entangled and interconnected structures, indicating that small alterations in the sequence can cause significant changes in the structure of resulting gels.

Peptide amphiphiles (PAs) are another class of self-assembling building blocks for hydrogel formation. PAs can be of three subclasses: (1) amphiphilic peptides; (2) lipidated peptides and (3) PAs conjugated with supramolecular binding motifs [42].

Amphiphilic peptides are composed of amino acids only. The balance between hydrophobic and hydrophilic forces largely contributes to the self-assembly process of amphiphilic peptides. A pH-responsive supramolecular peptide hydrogel was self-assembled from a synthetic peptide called PEP-1 (Ac-FALNLAKD-NH_2_) [43]. In the PEP-1 sequence, F, A and L amino acid residues are hydrophobic while D, N and K are hydrophilic, making PEP-1 an amphiphilic peptide. PEP-1 was able to form hydrogel at pH 7.4 due to the electrostatic interaction between aspartic acid (D) and lysine (K) residues, but the structure could be destroyed either in acidic or basic environments (pH 5.5, 9.0 and 12.0). In acidic environment, the protonation of the carboxylates in aspartic acid was not able to hold the electrostatic interaction with lysine amine groups and keep the entangled nanofibers, while in the basic environment, the increased solubility of PEP-1 and electrostatic repulsion between aspartic acid residues may be responsible for the lack of well-defined assembly.

Lipidated peptides are hybrid molecules consisting of a hydrophobic alkyl (lipid) tail and a peptide segment containing, or not, sequences to form secondary structures, and a hydrophilic head to enhance water solubility. This class of PAs have been widely reported in the literature due to their design versatility and diversity of self-assembled nanostructures [44]. As such, they offer great potential to create a range of biomaterials for different biomedical applications, from drug delivery to TE [45]. Many PAs are designed to contain a β-sheet forming segment in order to promote their self-assembly into nanofiber structures. An injectable hydrogel was prepared based on palmitoyl-GNNQQNYKD-OH PA. Incorporation of the triptolide drug did not affect the hydrogel formation [46].

PA conjugates, consisting of PA molecules bearing supramolecular motifs at the C-terminus were recently reported to enable noncovalent cross-linking between PA nanofibers (Figure 3b). β-CD and Ad were coupled to a cationic PA (palmitoyl-V_3_A_3_K_3_), separated by a glycine spacer (G_3_), by copper(I)-catalyzed alkyne−azide cycloaddition [21]. The resulting supramolecular hydrogel showed enhanced mechanical properties and resistance to degradation. Hydrogels formed by PA-DNA conjugate nanofibers cross-linked by DNA hybridization were also reported by the Stupp group [47]. Oligonucleotides were covalently linked to a lysine side chain at PA C-terminal by click chemistry to obtain PA-DNA conjugates, which was then co-assembled with a filler PA. Their co-assembly at different molar concentrations results into nanofibers displaying single-stranded DNA at different densities. Mixing fibers containing complementary DNA strands generates a reversible hydrogel which could disassemble when soluble single-stranded DNA is added as consequence of the toehold-mediated strand displacement mechanism. The dynamic organization of the nanofibers within the hydrogel network was shown to modulate phenotypic transformations in astrocytes.

Selection of supramolecular hydrogels using polymer or peptide building blocks requires some considerations from the development and the application point of view. We have attempted to identify advantages and disadvantages associated with both types of hydrogels (Table 2).

### 2.3. Nucleic Acid-Based Hydrogels

Although not widely exploited as polymers and peptides, nucleic acids (mainly DNA) are gaining significant attention as building blocks for the supramolecular fabrication of hydrogels. Hydrogels can be formed by reversible cross-linking through DNA self-assembly (two complementary single-stranded DNA molecules can form a single double-stranded molecule through Watson-Crick base pairing, a process known as DNA hybridization) and can consist entirely of DNA or short DNA sequences grafted onto polymer backbones. Furthermore, using protein-binding aptamers, proteins can be captured within the DNA-based hydrogel (Table 3) and their release initiated using the displacement strand strategy. However, the use of DNA strands as the release trigger may not feasible in in vivo applications. Two recent reviews provide insightful background on the design, properties and biomedical applications of supramolecular DNA-based hydrogels [48,49] and thus this type of hydrogels will not be discussed in detail here.

The formation of hydrogels using nucleopeptides was reported by Xu and collaborators where nucleobases (thymine, adenine, cytosine, and guanine) were conjugated at the *N*-terminus of short peptides (FF, FFY, FFY_p_) [50]. The nucleopeptides were able to self-assemble in water, upon a pH- or enzyme trigger, and were shown to be resistant to proteinase K, a proteolytic enzyme. The self-assembled nucleopeptide hydrogels supported cell migration. Following a similar conjugation approach, the group of Laura Suggs screened a nucleo-tripeptide library for their ability to form hydrogels at physiological conditions [51]. The mechanical properties of the hydrogels varied from 10 Pa to 1 kPa depending on the nucleobase and amino acid composition. Oligonucleotides (length of 19 bases) have been also conjugated at the C-terminus of the self-assembling Fmoc-FF-OH peptide using copper-free click chemistry to yield pepDNA_19_ [52]. Mixing peptides bearing complementary oligonucleotides promoted nanofiber bundling which could lead to gel formation. PepDNA_19_ assemblies were sensitive to pH changes and could be degraded by DNase. More recently, the impact of C-terminus chemistry on the self-assembly of guanosine (gs)-containing nucleopeptides (gs-GKFF) was investigated [53]. The self-assembly was governed by the peptide segment, forming β-sheet structures, with the hydrogen-bonded guanosine (G-quartet or G-ribbon) contributing with additional secondary structures within the peptide conformation. The morphologies of the nucleopeptides assemblies were shown to depend on the C-terminus chemistry (amide or carboxylic acid).

Combining nature’s building blocks in a single molecule, a nucleobase (thymine, cytosine, adenine, or guanine) linked to an amino acid (one or two phenylalanine) and glycoside (d-glucosamine), Xu’s group designed a new class of supramolecular hydrogelator [54], which were shown to self-assemble in water and form hydrogels at concentration at 3 wt.%. The hydrogels exhibited viscoelastic properties, reaching storage modulus of 220 kPa, and stability in presence of proteolytic enzymes. They were able to bind and deliver nucleic acids to cells. By expanding the range of building blocks for fabricating supramolecular hydrogels, novel functional materials with new properties can be discovered for applications in TE.

### 2.4. Multi-Component Hydrogels

More recently, combining peptides with either natural or synthetic polymers has resulted in a new class of hybrid supramolecular hydrogels aiming to improve their mechanical properties and/or improve their biological or chemical responsiveness [55]. In general, hydrogels fabricated by peptides on their own present poor mechanical properties [56]. At the same time, peptides are able to enhance the gelation process, preventing polymer aggregation [57], as well as providing a source of therapeutic molecules.

In this section, we defined “hybrid” as a multi-component supramolecular hydrogel formed via physical interaction between polymers and peptides or modified peptides, instead of self-assembly of hybrid lipopeptides, such as PAs. One of the earliest report on the formation of hybrid supramolecular hydrogels is the self-assembly of a heparin-binding PA with heparin reported by the Stupp group [58]. Gel formation was attributed to the electrostatic interaction between the negatively charged heparin chains and the positively charged PA with the sequence of palmitoyl-AAAAGGLRKKLGKA. The PA molecule presented an initial α-helix structure, but changed to β-sheet conformation after addition of heparin, which contributed to the PA self-assembly into nanofibers and gel formation. 

**Figure 5:**
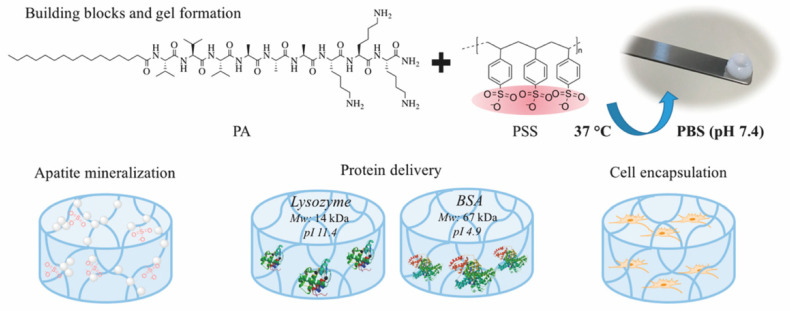
Representative multicomponent supramolecular hydrogel self-assembled between PA and PSS. The PA/PSS hydrogel is multifunctional providing sites for calcium phosphate mineral nucleation and a hydrated network for protein delivery and 3D cell encapsulation. Adapted with permission from [61] Copyright © (2019), American Chemical Society.

Electrostatic interaction is the most popular driving force to form hybrid supramolecular hydrogels. *N*-fluorenylmethoxycarbonyl diphenylalanine (Fmoc-FF-OH) and poly-l-lysine (PLL) were gelled together to form an injectable supramolecular hydrogel [56]. Fmoc-FF-OH is able to self-assemble into β-sheet structure but with poor rheological properties. When combined with PLL, the electrostatic interactions between positively charged PLL and negatively charged Fmoc-FF-OH nanofiber result in an enhancement of mechanical properties. Furthermore, thiol groups were introduced into PLL to improve the stability of hydrogels. Through various interactions, such as hydrophobic interaction, electrostatic forces, π-π stacking and hydrogen bonding, the PLL-SH/ Fmoc-FF-OH hydrogel could present nanofibers in helical conformation with amphipathic and amphoteric behavior. Borges et al. [59] reported a hybrid peptide/polymer supramolecular hydrogel combining self-assembly and layer-by-layer (LbL) assembly technique. Low molecular weight PA with sequence lauryl-VVAGKKK-NH_2_ (K_3_PA) was synthesized consisting of a hydrophobic lauryl tail, a hydrogen bonding sequence and a positively charged hydrophilic sequence. This PA was able to interact with anionic high molecular weight alginate (ALG) biopolymer via electrostatic interaction. Then, quartz crystal microbalance with dissipation monitoring technique was applied to study the nanofilm build-up process. A longer adsorption time was needed for the deposition of K_3_PA molecule compared with ALG, showing that the binding and arrangement of K_3_PA was slow. Although not reported in this study, multiple GFs could be loaded during multi-layer build-up for sequential GF co-delivery [60]. Using the negatively charged synthetic polymer poly(sodium 4-styrenesulfonate) (PSS) and a positively charged PA (palmitoyl-V_3_A_3_K_3_-NH_2_), a hybrid supramolecular hydrogel was reported recently (Figure 5) [61]. Upon mixing PA with PSS, self-supporting opaque hydrogels were formed within minutes. Rheology tests demonstrated the formation of stiff PSS/PA hydrogels and their stiffness and stability could be tuned by adjusting the chain length of PSS.

The self-assembly of nucleopeptides with single-stranded DNAs (ssDNAs) into hydrogels at physiological pH (pH 7.4) has been reported by Xu and colleagues [62]. To enable interactions between the nucleopeptide and ssDNA, three nucleobases (two thymines and one cytosine) were coupled to ε-amine on the lysine side chains of the peptide Nap-FFKGKGL-OH. The nucleopeptide formed a weak nanofiber gel on its own, but the addition of ssDNAs induced nanofiber bundling and contributed to the formation of a stronger hydrogel.

An injectable hybrid hydrogel fabricated by an amphiphilic small peptide (Fmoc-FF-OH) and a fullerene derivative called C_60_ pyrrolidine tris-acid (C_60_-PTC) was reported [57]. Fmoc-FF-OH itself could self-assemble into a β-sheet nanofibrous transparent hydrogel. Upon integration of C_60_-PTC, the β-sheet structure changed to α-helix, which mainly resulted from hydrogen bonding together with electrostatic repulsion between Fmoc-FF-OH and C_60_-PTC. C_60_-PTC appeared as uniform nanoparticles with diameter of 6 nm instead of the aggregates (110 nm) as observed in water, indicating that the hydrogen bonding and electrostatic repulsion between Fmoc-FF-OH and C_60_-PTC inhibited the hydrophobic and π-π interactions between C_60_-PTC molecules. The broadened bands from UV-vis absorption spectra suggested that hydrophobic and π-π interactions between C_60_-PTC also contributed to the hydrogel formation. As a result, mechanical properties were improved and the ^1^O_2_ generation activity of fullerene was enhanced due to the uniform distributed C_60_-PTC. This led to improved wound healing due to the antibacterial effect of sustained reactive oxygen species production.

## 3. Protein Loading and Release from Supramolecular Hydrogels

Drug delivery systems not only provide protection of entrapped molecules against degradation, but also offer the possibility to control their release at desired sites and rates to achieve maximum therapeutic effect. The application of supramolecular peptide hydrogels for the delivery of protein drugs and other biologics has been recently reviewed [63,64], demonstrating the versatility of this type of hydrogels for the controlled release of a variety of protein therapeutics with relevance in TE. Polymer and DNA-based supramolecular hydrogels have been also widely used for the controlled release of proteins while the application of nucleopeptide-based hydrogels has not been exploited yet despite they were shown to provide sustained release doxorubicin [65].

**Figure 6:**
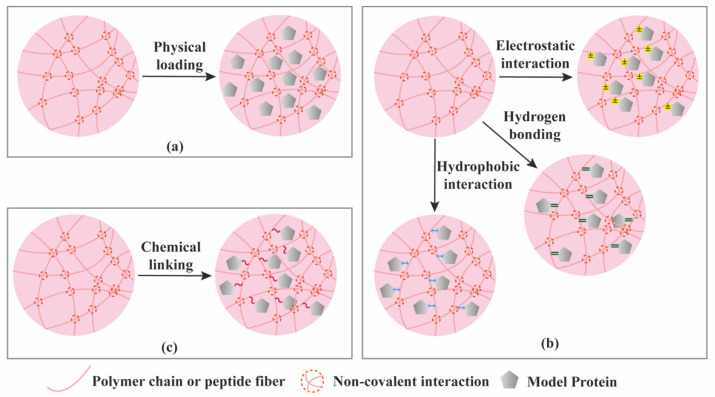
Methods for loading proteins into supramolecular hydrogel networks. (**a**) Proteins are physically loaded in the hydrogel network; (**b**) Proteins establish non-covalent/affinity interactions with hydrogel network; (**c**) Proteins are chemically linked to the hydrogel network.

**Figure 7:**
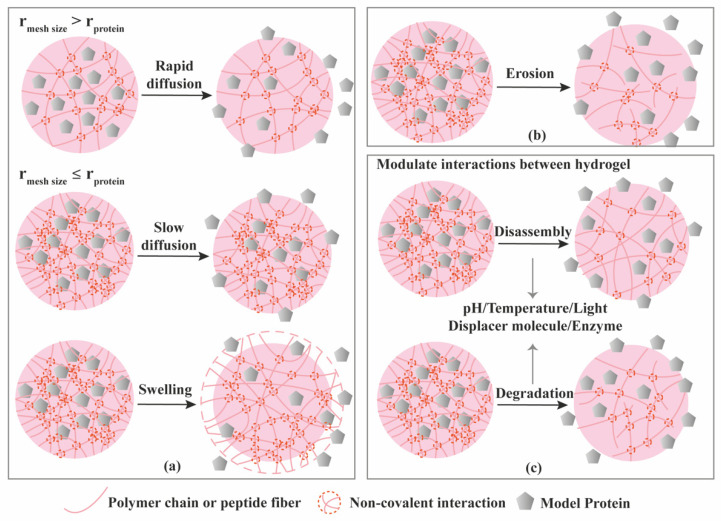
Different release mechanisms from supramolecular hydrogels. (**a**) Diffusion-controlled release; (**b**) Erosion-controlled release; (**c**) Stimuli-controlled release by modulating the hydrogel network.

**Figure 8:**
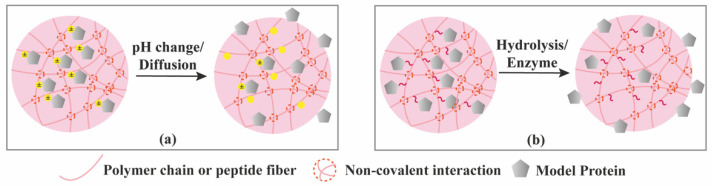
Controlled release of immobilized protein from supramolecular hydrogels by modulating the protein-hydrogel interactions. (**a**) pH changes can induce alterations in the protein or hydrogel charge favoring protein release. (**b**) Gradual hydrolysis or enzyme cleavage of bonds linking proteins to the hydrogel network results in the controlled release of protein over time.

Proteins can be loaded into hydrogels via three different ways (Figure 6): (1) Proteins are physically entrapped in the hydrogel network; (2) Proteins establish non-covalent/affinity interactions with hydrogel components; (3) Proteins are linked to the hydrogels via covalent bonds [66]. As a result of the various loading modes, different release mechanisms are expected (Figure 6, Figure 7 and Figure 8). Model proteins with different molecular weights and isoelectric points (pI), such as bovine serum albumin (BSA, 66.5 kDa, pI 5.3), lysozyme (14.3 kDa, pI 11.4), Immunoglobulin G (IgG, 150 kDa, pI 7.2) and soybean trypsin inhibitor (20.1 kDa, pI 4.6) [67], have been widely used to investigate the effect of size and charge on protein release from hydrogels, and optimize loading, before using the more expensive proteins with interest for TE applications, such as GFs. Table 3 summarizes the release mechanisms from supramolecular hydrogels using different model proteins.

### 3.1. Diffusion-Controlled Release

Hydrogels are typically characterized for their mechanical strength, mesh size, and swelling properties [29]. If the protein does not have affinity to the hydrogel components, and the mesh size of the hydrogel is larger than the hydrodynamic radius (r) of the protein, diffusion will become the leading driving force for the protein release, as depicted in Figure 7a. Mesh size is the size of open spaces between polymer chains which could be manipulated through the crosslinking density. On the contrary, if the mesh size is smaller than the hydrodynamic radius of the protein, the protein will be locked in the hydrogel network. Some hydrogels undergo volume change upon swelling, in which the hydrogel takes up water and swells. When the swelling occurs, the mesh size increases, resulting in rapid diffusion through the hydrogel (Figure 7a). We will not introduce swelling-controlled release separately, since the essence of this release mechanism is still diffusion through relatively larger mesh sizes. Up to now, many of the gel matrices are reported to exhibit diffusion-controlled release, following Higuchi’s kinetics, implying that the release is proportional to the square root of time.

MAX1 (VKVKVKVK-V^D^PPT-KVKVKVKV-NH_2_) and MAX8 (VKVKVKVK-V^D^PPT-KVEVKVKV-NH_2_) are self-assembling peptides that could form hydrogels with different mesh size via electrostatic interactions at physiological buffer conditions (pH 7.4, 150 mM NaCl) by changing their concentration [72]. Dextran with different molecular weights (20, 70 and 150 kDa), corresponding to different hydrodynamic diameters, were entrapped in the hydrogels to study the effect of mesh size on their release. Self-diffusion studies using fluorescence recovery after photobleaching and release studies demonstrated that the release profile of encapsulated macromolecules can be modulated by altering the mesh size of the hydrogels. In addition, lactoferrin, with different charge from dextran, was also loaded in the hydrogels to study the effect of charge on release. The release results proved that attractive electrostatic interaction retarded the release while repulsive electrostatic interaction enhances the release. Using different model proteins (lysozyme, IgG, lactoferrin, α-lactalbumin, myoglobin and BSA) loaded in MAX8 hydrogels also demonstrated the effect of charge on the release patterns [73]. A similar study was also carried out using positively charged HLT2 (VLTKVKTK-V^D^P^L^PT-KVEVKVLV-NH_2_) and negatively charged VEQ3 (VEVQVEVE-V^D^P^L^PT-EVQVEVEV-NH_2_) peptide hydrogels to demonstrate the effect of charge on protein release (Table 3) [74].

A self-gelling hydrogel, physically crosslinked by oppositely charged dextran microspheres, was obtained through ionic interactions using dex-HEMA-MAA (anionic microsphere) and dex-HEMA-DMAEMA (cationic microsphere). Three model proteins (IgG, BSA and lysozyme) were loaded and their release studied in vitro [68]. Confocal images showed lysozyme, with smallest Mw and positive charge at neutral pH, penetrated into negatively charged microspheres, while BSA, with negative charge but relatively higher Mw, was not able to penetrate into neither the negatively nor positively charged microspheres, but was able to adsorb onto the surface of positively charged microspheres. By contrast, IgG, with neutral charge, showed reduced adsorption. The results of in vitro release showed the release of all three proteins is governed by diffusion depending on their size and surface charge. Proteins with smaller hydrodynamic radius, like lysozyme, diffused faster since they are able to penetrate the microsphere to reach the surface of hydrogel directly, while proteins with larger hydrodynamic radius, like BSA and IgG, must bypass the microspheres and thus longer time is required.

The influence of polymer concentration on the release of entrapped proteins was studied using a host-guest self-assembled hydrogel [69]. Hydrogels with different polymer concentrations (0.5 wt.% and 1.5 wt.%) were prepared from a poly(vinyl alcohol) polymer modified with viologen (PVA-MV, first guest), a hydroxyethyl cellulose functionalized with a naphthyl moiety (HEC-Np, second guest), and cucurbit [8] uril (CB [8], host), and then loaded with either BSA or lysozyme. At relatively higher polymer concentration, more sustained release was achieved and burst release was suppressed, independently of the protein type. Generally, the polymer concentration directly influences the crosslinking density of the hydrogel and subsequently the mesh size [6]. The mobility of proteins in a dense hydrogel network is reduced, contributing to a sustained release.

Using the established Ac-(RADA)_4_-CONH_2_ peptide hydrogel, protein release was studied using various model proteins including lysozyme, trypsin inhibitor, BSA and IgG [67]. Release kinetics and diffusion coefficients were determined by single-molecule fluorescence correlation spectroscopy method which could also calculate the diffusion coefficients of the protein inside the hydrogel during the release process. The hydrogel was able to carry a high protein load, hence molecular crowding inside the hydrogels should play a role in protein diffusion. In addition, hydrogel density and conformation of proteins should also not be excluded. Release data plotted as a function of the square root of time showed that the diffusion mechanism of all the four model proteins is biphasic. The initial linear part indicated diffusion-controlled release while deviation from the straight line at longer times might be associated with anomalous diffusion.

### 3.2. Erosion-Controlled Release

Erosion-mediated release has been exploited in supramolecular hydrogels for the controlled delivery of proteins that is regulated by degradation of the hydrogel structure (Figure 7b). A self-healing supramolecular hydrogel formed with the N4-octanoyl-2′-deoxycytidine gelator was used to encapsulate several proteins (BSA, β-lactoglobulin, lysozyme and insulin) and study their release profile [78]. The release profiles of all the model proteins exhibited similar trends within 24 h and were also consistent with the profile of hydrogel degradation, suggesting that degradation might be the dominated mechanism for release regardless of the properties of loaded proteins. Then, real time fluorescence microscopy was used to follow the release of Cy5-BSA from the supramolecular hydrogel. After 24 h, the bulk hydrogel had been fully eroded leaving smaller fluorescent fragments floating in the release medium. The results from fluorescent images, together with the protein release data, demonstrated that the release of encapsulated proteins followed the erosion of the supramolecular hydrogels.

In vivo release studies were conducted for a peptide-based supramolecular hydrogel [75]. The hydrogel was formed by self-assembly of the amphipathic hexapeptide H-FEFQFK-NH_2_, and three kinds of model cargos were encapsulated including a small molecule, a 15-residue peptide and a small protein. The cargo molecules were labelled with radioactive isotope ^11^In and loaded in the hydrogels. The prepared hydrogels were subcutaneously injected into a mouse model and the release in vivo was investigated by SPECT/CT imaging. The small molecule was released in a rather fast manner which may be caused by diffusion due to the small size, while the peptide and protein showed a similar release profile with sustained release up to 12 h. The in vivo stability of the hydrogel was also monitored by incorporation of radioactive isotope ^11^In labelled peptide hydrogelator. The volume of hydrogel was measured by detecting the radioactive signal remained at the injection site. The hydrogel presented nearly 75% degradation in the first 12 h after injection, which is consistent with the release profile of the cargo, indicating that the observed cargo release is linked with the degradation of the supramolecular hydrogel.

### 3.3. Stimuli-Controlled Release

Generally, stimuli-responsive hydrogel delivery systems include stimuli-sensitive units to enable network changes upon stimulus trigger (Figure 7c). Different physical, chemical and biological signals have been used as stimuli, including temperature, pH, enzymes, light, magnetic and electric fields. The stimuli act as a switch to control the hydrogel network at desired time and location which enable a precisely controlled release.

Temperature-responsive supramolecular hydrogels, using thermo-sensitive hydrogelators as the building blocks, are the most widely studied systems [82]. A synthesized amphiphilic copolymer Py-PCL-*b*-POEGMA was able to self-assemble into micelles at room temperature and subsequently form a supramolecular hydrogel through the host–guest interaction between α-CD with POEGMA [33]. In vitro release showed that loaded BSA presented a faster release at 37 °C compared to 25 °C. This results from the dissociation of α-CD when the temperature is increased. Therefore, the hydrogels suffered partly from structural damage. Moreover, when the temperature goes up, protein molecules will present higher mobility which also contributed to the faster release at 37 °C.

Using pH as a stimulus is another effective way to create smart supramolecular hydrogels since pH could significantly adjust the intensity and strength of hydrogen bonds as well as the ionic forms of amphiphilic gelators only with a small shift. As an example, Wang et al. [76] reported a pH-switchable supramolecular hydrogel from a designed octapeptide Ac-IKFQFHFD-NH_2_. The peptide self-assembled into nanofibers at neutral pH (pH 7.4) and disassembles at acidic pH (pH 5.5) due to the protonation of carboxylate group of aspartic acid (D), making pH-controlled release of the peptide hydrogelator in acidic wound environment and generate antimicrobial effect.

Enzyme-triggered release is also very appealing for TE applications since enzymes regulate many reactions in tissues. Hydrogels formed by natural polymers are subject to the action of a variety of enzymes such as dextranase, elastase, hyaluronidase, and matrix metalloproteinases (MMPs). Enzymatic cleavage of certain peptide sequence was also used as a strategy to achieve hydrogel degradation [83]. Ac-I_3_SLKG-NH_2_ is an amphiphilic peptide able to self-assemble into fibrillar hydrogels [77]. This peptide hydrogel was able to be degraded into Ac-I_3_S-OH and H-LKG-NH_2_ in response to MMP-2. When an anticancer peptide G3 with sequence of G(IIKK)_3_I-NH_2_ was loaded into the hydrogels, its release was revealed to be enzymatic responsive when MMP-2 was added. Similar hydrogel design could be applied for the delivery of GFs to promote bone regeneration as increased levels in MMP-2 protein expression have been detected in the fracture callus during bone fracture repair [84]. Two diphenylalanine derived peptides with acetoxybenzyl-oxycarbonyl (APmoc-F(CF_3_)F-OH) or benzoate (Bz-FF-OH) groups tethered at the N-terminus were synthesized to obtain enzyme-sensitive supramolecular hydrogels [79]. Cleavage of N-terminal moiety decreased the hydrophobicity leading to the gel-sol transition. Bovine carbonic anhydrase II (bCAII) is an enzyme able to cleave the protecting group at the N-terminus in the APmoc-F(CF_3_)F-OH leading to a gel-sol transition. Its activity can be inhibited by an enzyme-activity trigger (EAT), consisting of a bCAII inhibitor linked to biotin, a strong ligand of avidin. bCAII and EAT were mixed with APmoc-F(CF_3_)F-OH before gelation. Gel-sol transition was observed after avidin was added to the system and incubated for 6 h. However, the hydrogel remained in its gel state if avidin was added together with biotin. This phenomenon revealed that EAT preferentially bound to avidin because of steric repulsion, leading to activity recovery of bCAII and resulting in the degradation of the hydrogels. Then, APmoc-F(CF_3_)F-OH hydrogel was mixed with agarose to produce a supramolecular/polymer composite hydrogel in order to improve mechanical properties and protein entrapment. Myoglobin (Mb), used as model protein, was loaded into the composite hydrogel to study the enzyme-controlled release. 75% of Mb was released after the addition of avidin, while only 2.3% of Mb was released if incubated only in buffer, showing an enzyme-controlled release. This enzyme-sensitive hydrogel can work as a non-enzymatic protein-responsive protein release system, which could be applied to trigger GF release by a biomarker protein.

As mentioned in Section 2 and Section 3, light can act as a precise and well controlled external stimulus by including light-sensitive groups in the hydrogel network. The transition of hydrogel network upon light irradiation achieves control over drug release [17]. FITC-BSA was encapsulated in HA-β-CD/HA-Azo hydrogels and upon irradiation with ultraviolet light (365 nm), hydrogels released over twice as much protein as the nonirradiated hydrogels, which revealed that the hydrogel disassembles under irradiation allowing for cargo leakage. After removal of light stimulus, the release profile of irradiated hydrogel had a similar trend with that of the nonirradiated one, showing good light responsiveness.

Many supramolecular hydrogels described above can exhibit combined release kinetics. For example, in the absence of external/internal stimuli, slow diffusion is the dominant mechanism followed by burst release when stimuli are applied [17].

### 3.4. Chemical Interactions-Mediated Release

Bioactive proteins can be immobilized into hydrogels by creating hydrogen bonding, hydrophobic or electrostatic interactions between the hydrogel network and the protein. In the absence of stimuli, proteins will slowly diffuse from the hydrogel, but electrostatic interactions can be modulated by pH changes (Figure 8a) and thus promoting their release. To ensure long-term release, proteins can be covalently tethered (or fused) onto the hydrogel network (Figure 8b). However, bioactive proteins, such as GFs, typically exert their activity by binding to their corresponding receptors, requiring a certain level of mobility to reach their target binders. As such, the linkage should be prone to hydrolytic or enzymatic cleavage in order to release the attached protein. Chemical linkages can be permanent or cleavable. In the first case, the attached protein is released when the hydrogel network degrades (Figure 7b or Figure 7c), while in the second case specific cleavable linkages can be broken down over time by hydrolysis or in presence of certain environmental stimulus such as enzymes [6]. For example, the release of fluorescent functional proteins (GFP, YFP) covalently attached to the DNA crosslinker in protein-DNA hybrid hydrogels (Table 3) was achieved by enzymatic degradation of the protein network (trypsin) or cleavage of DNA backbone (DNase) [80].

## 4. Supramolecular Hydrogels for the Delivery of Bioactive Proteins for TE Applications

By modulating cell’s behavior, GFs play an important role in activation of cascades to regenerate damaged tissues [2]. However, GFs are generally unstable in physiological conditions and are degraded by enzymes in a very short time, so frequent and high-dose injection of GFs is required to achieve therapeutic effects [85,86]. In addition, GFs are a group of multifunctional bioactive proteins, which may bind to different GF receptors and generate different effects [3]. Therefore, controlled and local delivery of GFs is key to harness their biological activity. Hydrogels are widely used to achieve precise delivery and controlled release of water-soluble molecules due to their high water content, soft nature and porous structure [66]. In this section, some recent studies on applications of supramolecular hydrogels for the delivery of GFs in TE will be described.

### 4.1. Vascular Tissues

Vascularization is key in tissue regeneration by providing adequate oxygen and nutrients to ensure the normal function of tissues. Therapeutic vascularization is thus essential in TE strategies.

Angiogenesis is a process regulated by various GFs to form new blood capillaries from small existing vessel wall. Vascular endothelial growth factor (VEGF) is an essential GF that regulates the proliferation and migration of endothelial cells to initiate angiogenesis process. However, the in vivo half-life of VEGF is very short, approximately 50 min [87], requiring methods for its effective delivery.

RAD16-I peptide was combined with heparin to form multi-component supramolecular hydrogel [88]. The presence of heparin improved the binding of several GFs such as VEGF_165_, TGF-β1 and FGFβ. Release studies showed that the release of bound GFs was slower than from the RAD16-I hydrogels without heparin. Moreover, the biological effect of released VEGF_165_ and FGFβ was examined by culturing human umbilical vein endothelial cells (HUVECs) in the release media. Cell viability results showed a significant effect of the released VEGF_165_ and FGFβ on HUVECs maintenance and proliferation with higher live cell numbers compared to the control where almost all cells were dead, demonstrating that the biological activity of the GFs was maintained in the hydrogels.

Recently, the use of an injectable silk fibroin (SF) hydrogel combined with a peptide-based gelator for the local VEGF delivery was reported [89]. SF hydrogels were shown to be good candidates for TE but have very slow dynamics of gelation, usually more than 5 days. A known biocompatible peptide gelator was chosen to accelerate the gelation process instead of the traditional sonication, pH adjustment, or the addition of organic molecules. NapFF-OH, containing a naphthyl group and the FF dipeptide, self-assembles into nanofibers in solution at a low concentration, while in the solution of SF, the one-dimensional nanofiber could interpenetrate with SF resulting in a three-dimensional nanofibril network. To improve cell adhesion ability in vivo, an RGD modified peptide gelator (NapFFRGD) was synthesized to replace NapFF-OH and form a new supramolecular hydrogel with SF (Gel RGD). VEGF was encapsulated in the gel containing RGD to provide angiogenesis therapy. VEGF-loaded Gel RGD was implanted subcutaneously in a mice model. The increased density of blood capillary could be observed only 3 days later after implantation. Furthermore, immunohistochemical staining with CD31 antibodies demonstrated that endothelial cells migrated and grown on the implanted gel matrix, suggesting the Gel RGD not only served for the delivery of VEGF but also provided a suitable microenvironment for efficient vascularization.

### 4.2. Bone

**Figure 9:**
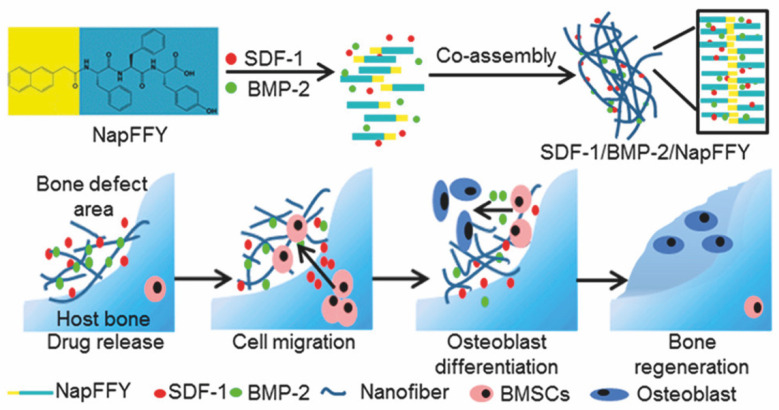
Schematic illustration of the formation process of the SDF-1/BMP-2/NapFFY hydrogel and mechanism for periodontal bone regeneration in the bone defect area. Slow SDF-1 release recruits BMSCs to the defect area and released BMP-2 promotes BMSCs differentiation into osteoblasts, resulting in the initiation of the periodontal bone regeneration process. Adapted with permission from [90] Copyright © (2019), American Chemical Society.

Strategies in bone TE often use biomaterials for local delivery of stem cells and bioactive factors, which are essential for inducing stem cell differentiation and bone growth. A supramolecular hydrogel prepared by Nap-FFY-OH was established to co-deliver stromal cell derived factor-1 (SDF-1) and BMP-2 and promote periodontal bone regeneration [90] (Figure 9). SDF-1 is a chemokine known to promote the recruitment and proliferation of BMSCs and periodontal ligament stem cells (PDLSCs), while BMP-2 induces the differentiation of BMSCs. Different from the above design, the two GFs were used to recruit BMSCs in situ instead of exogeneous delivery, which may have low survival rates. As shown in Figure 9, SDF-1, BMP-2 and NapFFY were able to form nanofibers (SDF-1/BMP-2/NapFFY) with a diameter of 44.6 nm when simply mixing them together. A constant and sustained release up to 35 days was achieved in vitro with a total release amount of 74.8% for SDF-1 and 82.1% for BMP-2, which is a suitable release period for GFs in bone regeneration applications. Chemotactic effect of SDF-1/BMP-2/NapFFY hydrogels in transwell culture on BMSCs showed the release of SDF-1 and BMP-2 were able to induce chemotaxis and differentiation of BMSCs, respectively. In vivo bone regeneration was assessed using the critical-sized periodontal bone defect model of maxillae in rats. SDF-1/BMP-2/NapFFY hydrogels accelerated bone bridging and defect reunion processes compared with single drug groups, indicating co-delivery of SDF-1 and BMP-2 by NapFFY hydrogels presented a synergistic effect for bone growth. More importantly, the supramolecular NapFFY hydrogel also provided an ECM-like microenvironment suitable for adhesion and growth of BMSCs and PDLSCs, further supporting the function of stem cells.

Bone and cartilage form bone-cartilage interface to help the body movement. It is worth noting that intimate integration between different tissues is very important for the function recovery when the tissue complex is damaged [91]. Supramolecular hydrogels typically have self-healing properties, making them suitable for the repair of such tissue complexes. A self-integration and shear-thinning hydrogel was produced to facilitate delivery at the bone-cartilage complex [92]. The supramolecular hydrogel was self-assembled by Ureido-pyrimidinone grafted dextran (DEX-UPy) when the UPy modification was sufficiently high. UPy is a synthesized multi-hydrogen-bonding polymer which could provide higher intermolecular hydrogen bonding. The self-healing ability of DEX-UPy hydrogel was tested by cutting the formed hydrogel into pieces, labeling with a colored dye and placing the pieces back together. Interestingly, the separated hydrogel integrated together within minutes after contact. The rapid re-formation was likely to result from the hydrophobic segments and urea group, which stabilized the nanofiber formation. Chondrocytes for cartilage formation and bone marrow stem cells (BMSCs) together with bone morphogenetic protein 2 (BMP-2) were encapsulated in the separated parts of the hydrogel, and the two parts were then let to achieve self-integration. The integrated hydrogels were subcutaneously implanted in nude mouse model to test their ability for osteochondral tissue regeneration. 8 weeks later, the positive results of histological staining demonstrated the growth of both cartilage and bone tissues in their spatially defined regions with seamless connection.

A self-assembling PA system able to bind both endogenous and exogenous BMP-2 was reported in order to reduce the therapeutic dose for bone regeneration [93]. BMP-2-binding PA (BMP2b-PA) and diluent PA, were co-assembled in the same nanofiber. BMP2b-PA, consisting of a BMP-2-binding sequence at the PA N-terminus, showed BMP-2-induced osteoblast differentiation in vitro. When BMP2b-PA was mixed with diluent PA at the 1:1 ratio, a nanofiber hydrogel was formed. The bone regeneration was evaluated in a rat posterolateral lumbar intertransverse spinal fusion model and the nanofiber hydrogel was demonstrated to induce a 100% spinal fusion rate, only with 1/10 of the dose within collagen sponge (control) which may benefit from the prolonged retention of GF in the nanofiber hydrogels. Interestingly, 42% spinal fusion rate was observed in the nanofiber hydrogel without loaded BMP-2. It is likely that endogenous BMP-2 (pI 9.0) interacted with the carboxyl rich PA nanofibers via electrostatic attraction so that recruitment of endogenous BMP-2 effectively decreased the required therapeutic dose of exogenous BMP-2.

### 4.3. Cartilage

Mesenchymal stem cells (MSCs) are an important source of cells for cartilage regeneration as they can differentiate into chondrocytes when sustainably exposed to chondrogenic GFs. Hence, a gelatin-based injectable supramolecular hydrogel was reported to simultaneously deliver MSCs and chondrogenic factors, the small molecule kartogenin (KGN) or transforming growth factor β1 (TGF-β1), to provide a chondrogenic factor-rich environment for MSCs [94]. The gelatin-based supramolecular hydrogels (HGM hydrogels) were fabricated by host-guest interactions between the acrylated β-CD (Ac-β-CD) and the aromatic amino acid residues in gelatin. Hydrophilic TGF-β1 and MSCs were encapsulated directly in the hydrogels, and KGN, as hydrophobic molecule, was loaded in the non-occupied cavities of β-CD. A chemically crosslinked methacrylated gelatin hydrogel (GelMA) was also prepared for comparison. The release kinetics of KGN and the model protein BSA from HGM supramolecular and chemically crosslinked GelMA hydrogels were very different. KGN was released continuously for up to 28 days at a constant rate, but presented a fast release from GelMA within one week. BSA release was also slower in HGM hydrogels than in GelMA. The phenomenon was likely due to the host-guest structure acting as reservoirs of BSA molecules and improving the retention in HGM hydrogels. Then, chondrogenic differentiation of MSCs was examined both in vitro and in vivo. Expression of chondrogenic markers including aggrecan, type II collagen, SOX9 and the quantification of glycosaminoglycans (GAGs) were detected and all these markers exhibited significantly higher expression in HGM hydrogel-treated group than GelMA treated one, both in KGN and TGF-β1 encapsulated hydrogels, indicating that the HGM gelatin hydrogels promoted the chondrogenesis of the encapsulated MSCs. Finally, a rat osteochondral defect model was used to examine regeneration of cartilage defect. HGM and GelMA hydrogels were injected into the defective rat knee and allowed for 6 weeks before histological examination. In GelMA hydrogel-treated groups, little regeneration was found in the defect area. However, in HGM hydrogel treated groups, enhanced regeneration was observed with the formation of an organized osteochondral structure similar to normal tissue. The results indicated that sustained release of KGN or TGF-β1 provide sufficient chondrogenic factors for a long period of time which may contribute to chondrogenesis and ultimately cartilage regeneration.

HA and its derivatives have been widely explored for viscosupplementation of arthritic joints [95]. Similar “host-guest macromer” hydrogels (HGM hydrogels) were prepared using Ad modified HA (AD_x_HA, x denotes different modification degrees) to deliver MSCs and TGF-β1 [96]. Rats with osteochondral defects treated by cells and GF loaded in HA-based HGM hydrogels demonstrated considerable cartilage regeneration ability. MonoCB[6]/DAH-HA hydrogel is another HA-based supramolecular hydrogel used for cartilage regeneration [97]. MonoCB[6]/DAH-HA hydrogel was self-assembled by highly water soluble cucurbit[6]uril-hyaluronic acid (CB[6]-HA), diaminohexan conjugated HA (DAH-HA) and drug-conjugated CB[6] (drug-CB[6]). The purpose to prepare the monoCB[6]/DAH-HA hydrogels was also to promote chondrogenesis of MSCs in the presence of chondrogenic factors (dexamethasone and TGF-β3). Dexamethasone was conjugated to CB[6] (Dexa-CB[6]) with an ester bond for long-term sustained release after its modular modification to monoCB[6]/DAH-HA hydrogels. After hydrogel formation, the release profile of free dexamethasone, Dexa-CB[6], or TGF-β3 were first examined in vitro. Free dexamethasone without modification released rapidly from monoCB[6]/DAH-HA hydrogels within 2 h, while release was slower from Dexa-CB[6] lasting for more than 3 weeks. This is mainly due to the stable binding between Dexa-CB[6] and monoCB[6]/DAH-HA hydrogels. For TGF-β3, the release was able to last for a week which is much faster than Dexa-CB[6] because of physical entrapment. The in vivo differentiation of MSCs in MonoCB[6]/DAH-HA hydrogels was investigated in an animal model. Hydrogels were injected to the back subcutis of nude mice and analysed after 4 weeks. The expression level of GAGs in MonoCB[6]/DAH-HA group was higher than in the control groups, and chondrogenic markers, including COL II and SOX9, in cartilage tissue were all well expressed as observed from western blotting, indicative of chondrogenesis. Hence, monoCB[6]/DAH-HA supramolecular hydrogels have great potential for the synergistic effect of TGF-β3 and Dexa-CB[6] in the differentiation of MSCs, leading to effective chondrogenesis.

### 4.4. Skin

Wound healing is a complex process in which numerous factors, such as epidermal growth factor (EGF), fibroblastic growth factor-2 (FGF-2), are activated and released to accelerate the healing process. GFs are crucial in wound healing but they are susceptible to proteolytic degradation in the wound area. Hence, supramolecular hydrogels can be utilized to protect the GFs and maintain an adequate concentration.

EGF is a key factor involved in the wound healing process able to shorten the healing time by accelerating re-epithelialization through binding to EGF receptors [98]. An on-demand release of EGF was achieved by utilizing a photo-sensitive supramolecular hydrogel [99]. HA was selected as hydrogel network backbone, and conjugated with CD and Azo to form host-guest interaction. It is very easy to modulate the release from this supramolecular hydrogel with the control of UV irradiation. EGF was encapsulated in the hydrogel (EGF@PR-S gel) for local delivery and expected for on-demand release. A control supramolecular hydrogel, non-responsive to light, was prepared with Ad groups as guests (EGF@S gel). Both EGF@PR-S gel and EGF@S gel presented a typical 3D porous structure as observed by SEM. However, after 10 min of UV irradiation, the PR-S gel became soft and gradually conformed to the shape of the test tube while the S gel did not undergo any changes. When UV irradiation was removed, and the PR-S gel was exposed to visible light, the PR-S gel turned back to its stiffer state, confirming the photo-responsiveness of CD and Azo interaction. The release profile of EGF from those two hydrogels was monitored. When the hydrogels were exposed to the ambient light, EGF release from EGF@PR-S gels and EGF@S gels exhibited similar release profiles in a diffusion manner. However, when the hydrogels were exposed to UV irradiation, the EGF@S gel maintained its sustained release while EGF displayed a burst release from EGF@PR-S gel with approximately 2- to 3- times higher than that from EGF@S gels. In addition, when the irradiation was replaced by visible light, the release of EGF from EGF@PR-S gel decreased significantly to the previous level. This behavior showed that EGF release from EGF@PR-S gels could be easily modulated by alternating the irradiation. In vivo wound healing was assessed in an excisional full-thickness wound model in rats. Among the treated groups, the wounds treated with EGF@PR-S gel (with irradiation) showed the fastest recovery with almost complete wound closure, and the wound size showed over a 10% reduction compared with other treatment. The reason was likely due to the photo-triggered release of EGF at sufficient concentrations in the wound area. This research indicated the potential of photoresponsive supramolecular hydrogels to realize controlled, on-demand release of such bioactive agent.

The colonization of skin wounds by bacteria can create a cytotoxic wound microenvironment, delaying wound regeneration. Thus, a supramolecular hydrogel to combat wound damage as well as bacterial infection was established [100]. Silver ion (Ag^+^) was chosen not only due to its excellent broad-spectrum antimicrobial activity, but also for its interaction with chitosan (CS) through association of Ag ion with amino and hydroxyl groups in CS to rapidly form supramolecular hydrogels (CS-Ag hydrogels) at appropriate pH. To accelerate wound healing process, basic fibroblastic growth factor (bFGF) was encapsulated in CS-Ag hydrogels (bFGF@CS-Ag hydrogel) to stimulate the proliferation and migration of skin-related cells including keratinocytes, endothelial cells and fibroblasts. bFGF@CS-Ag hydrogel presented sol-to-gel transition within 1 min through association between Ag^+^ and amino and hydroxyl groups of CS at room temperature. A quick release of bFGF from bFGF@CS-Ag hydrogel was observed in the first day, followed by a sustained release lasting for more than 11 days, confirming a prolonged release of bFGF. Antibacterial effect was evaluated in vitro against both Gram positive and negative bacteria. Ag^+^ only presented the strongest antibacterial activity compared to the hydrogel groups. In vivo test was first carried out on an acute full-thickness wound model in mice. Interestingly, wound exposure percentage (an index to evaluate wound healing) showed no significant difference between bFGF@CS-Ag hydrogels treated group and bFGF or CS-Ag treated groups. However, H&E staining revealed the appearance of thick, newly formed granulation tissue after treatment with bFGF@CS-Ag hydrogels. Masson trichrome staining also showed more collagen deposition in the wound site in bFGF@CS-Ag hydrogel treated group than others, suggesting the pro-healing effect of bFGF@CS-Ag hydrogel. An infected wound model was also established to further test the wound healing ability of bFGF@CS-Ag hydrogel. The wound exposure percentage in bFGF@CS-Ag treated mice was the smallest with clean and closed wound, and the bacterial growth was effectively inhibited. This was likely attributed to the release of Ag^+^ which also induced the disintegration of the CS-Ag hydrogel, so that more bFGF was released to the wound site, showing a synergistic effect. The hydrogel degradation rate, and the corresponding release of metals ions from the hydrogel, may limit broader in vivo applications of such type of hydrogels due to the potential toxic effect in other tissues.

### 4.5. Others

In addition to the above applications targeting specific tissues, supramolecular hydrogels are also widely used in the regeneration of other tissues. For example, a polymer-based supramolecular hydrogel prepared from α-CD and methoxy polyethylene glycol-poly(caprolactone)-(dodecanedioic acid)-poly(caprolactone)-methoxy polyethylene glycol triblock polymer (α-CD/MPEG-PCL-MPEG) was used to deliver erythropoietin (EPO), a hormone reported to have a positive role in myocardial infarction (MI, to reduce the systemic side effect of thrombosis and hypertension [101,102]. A host-guest complex formed by CD modified hyaluronic acid (HA-CD) and Ad modified hyaluronic acid (HA-Ad) was prepared to co-deliver anti-TGF-β and anti-inflammatory cytokine interleukin-10 (IL-10) to treat chronic kidney disease (CDK) for localized immunotherapy to avoid renal fibrosis [103]. Table 4 summarizes the applications of supramolecular hydrogels to deliver proteins for the regeneration of different tissues. Overall, supramolecular hydrogels, with their self-healing and shear-thinning properties, controlled network density and stimuli behavior, have great potential for the local delivery of proteins with tailored release kinetics.

## 5. Challenges in the Design of Supramolecular Hydrogels

From the various studies described in this review, particular challenges arise for their clinical translation. Table 5 summarizes some of these challenges to be considered in the design of supramolecular hydrogels and proposes possible solutions to tackle them.

## 6. Clinical Challenges of Supramolecular Hydrogels

Protein drugs have gained increasing importance nowadays, including in TE applications. However, bolus injection of these biological molecules has shown low effectiveness due to their rapid elimination. Some GFs entering clinical trials have not shown the expected benefits to patients, while others have successfully passed through clinical trials. The application of a carrier system can further improve their clinical efficacy. For example, collagen sponges loaded with BMP-2 [107] and BMP-7 [108] are now commercially available to treat acute, open tibial shaft fractures by promoting growth of new bone at the site of implantation. The BMP-2 collagen sponge (INFUSE^®^ Bone Graft) is now undergoing clinical trial for the new indication of tibial pseudarthrosis in neurofibromatosis type 1, which is estimated to be completed in December 2021 (NCT02718131). However, these collagen sponges offer poor control over the protein release, requiring high GF doses. Supramolecular hydrogels, with their high water content, architecture similar with natural tissues, reversible transition in response to various stimuli, are promising carriers for controlled protein release. The first example of an injectable in situ forming hydrogel is an FDA approved thermo-sensitive hydrogel based on the assembly of a triblock copolymer of PLGA-PEG-PLGA, named ReGel^®^ [109]. ReGel^®^ was first used for delivery of paclitaxel, a chemotherapy drug for treatment of breast and esophageal cancer. Then, ReGel^®^ was extended for peptide or protein delivery, such as glucagon-like peptide (GLP-1) [110], insulin [111], BMP-2 [112] and interleukin-2 (IL-2) [113]. Although diverse supramolecular hydrogels as protein carriers have been developed in recent years, future efforts should be devoted to promote their clinical translation. The cohesive forces that hold supramolecular hydrogels are relatively weak compared to covalently crosslinked hydrogels. While this property enables their injectability, assessment of the sol-gel transition kinetics is crucial for determining their clinical application. Instant gelation may clog the needle before the end of the injection procedure. The use of hydrogels with low initial viscosity is preferred since it facilitates injection and decreases the risk of the hydrogel sticking in the needles. In addition, the ability to regain sufficient strength after injection stills needs to be investigated in more detail and optimized if necessary. Like for any biomaterial, hydrogel sterilization is required before their biological application. Conventional sterilization methods require elevated temperature, high pressure, radiation or use of sterilization chemicals. These methods are likely to cause disruption of the hydrogel structure and affect the protein activity. Donovan and coworkers found that ethylene oxide and gamma sterilization led to a reduction in the swelling fraction of a PEG-base hydrogel, while H_2_O_2_ sterilization increased the swelling fractions, affecting the release profile after sterilization [114]. A review article comparing sterilization methods for hydrogels has been published which can be used for guidance [115]. Considering that supramolecular hydrogels are formed in situ, it requires pre-sterilization of the hydrogel components and working under sterile conditions. Sterilization by filtration is commonly used for peptide and polymer solutions but careful evaluation needs to be done to ensure the final properties are maintained after sterilization. Stimuli-triggered release (e.g., pH, temperature, light) can promote protein denaturation and this needs to be considered when selecting the release trigger and its time-effect on protein structure and on the surrounding tissues. Some of the components used to fabricate the hydrogels are novel and require regulatory approval, posing additional challenges for their translation to clinical applications. For example, the degradation mechanism of some synthetic and natural polymers in vivo has not been fully elucidated. Hydrogels are generally designed to degrade into non-toxic soluble molecules which can be either metabolized or eliminated from the body [116], but this has not been demonstrated for all hydrogels described in this review. Finally, the scaling-up and reproducibility of supramolecular hydrogels need to be assessed in detail before they can be considered for clinical applications [64,117].

## 7. Conclusions

Delivery systems have been developed to increase the therapeutic outcome and reduce toxicity of drugs, while making therapies more cost-effective. Since proteins usually participate in multiple cellular pathways, their spatio-temporal controlled delivery is essential to direct them into desired pathways. As described in this review, supramolecular hydrogels, developed for over 30 years, offer a number of possibilities to deliver protein drugs with spatial (local delivery via injection) and temporal (via endogenous or exogenous triggers) control over their release. Because the hydrogel network is formed and held by non-covalent interactions, they enable the direct loading of sensitive protein molecules and their retention in the hydrogel via supramolecular interactions, not requiring additional chemical modifications of the protein drugs. Proteins contain charged and hydrophobic groups and can easily establish reversible associations with hydrogel components. The hydrated 3D-structure of supramolecular hydrogels enables high loading capacity to store therapeutic proteins. As such, supramolecular hydrogels can act as a depot of proteins, allowing their continuous supply locally or release when needed.

Even though supramolecular hydrogels offer numerous advantages as protein delivery systems for TE applications, important challenges remain to bring these systems into clinical application. Burst release is a common problem in hydrogel delivery systems, including supramolecular hydrogels, requiring further efforts to optimize the release profile. In addition, sustainable release of therapeutic proteins for several weeks in vivo via hydrogel delivery systems will demand innovative molecular engineering approaches to enhance their stability within the hydrogel for extended periods of time. Despite the recognized limitations and challenges with existing supramolecular hydrogels, their prospect in TE is very exciting, as demonstrated by the numerous and diverse examples described in this review.

## Figures and Tables

**Table 1:** Comparison between hydrogels with permanent covalent and reversible crosslinks in relation to criteria relevant for protein delivery.

Criterion	Covalently (Permanent Bonds) Crosslinked Hydrogels	Supramolecular Hydrogels
Processability	More complex; covalent bonds form during hydrogelation requiring additional reagents or inputs (e.g., light source).	Simple; it occurs spontaneously upon mixing hydrogel components or when in contact with electrolytes present in body fluids or culture medium.
Injectability	Difficult, unless if polymerizable in situ (e.g., under UV light).	Common in most hydrogels due to their shear thinning properties.
Self-healing ability	Absent, except if made of dynamic covalent bonds (e.g., disulfide).	Observed in most systems.
Protein loading	Mainly physical entrapment. Creating affinity interactions typically requires additional chemical modification of the hydrogel components.	Various possibilities (physical entrapment + affinity interactions) not requiring additional chemical modification.
Protein compatibility	Risk of protein deactivation during hydrogel formation if subjected to denaturing agents (e.g., catalysts, UV light).	Mostly compatible, unless there are strong electrostatic interactions between the protein and the hydrogel.
Stability	Stable at physiological conditions.	Less stable at physiological conditions (e.g., can be affected by differences in ionic strength).

**Table 2:** Pros and Cons of polymer- and peptide-based hydrogels.

Type of Hydrogels	Pros	Cons
Polymer-based	Great diversity of building blocks among synthetic and natural polymers Tunable mechanical properties via synthetic polymer (e.g., molecular weight, copolymer design) Good biostability Easily modified through a variety of functional groups available (e.g., carboxylic, hydroxyl) Easily controlled by stimuli	Potential toxicity of synthetic polymers Lack of bioactivity or biodegradability of some polymers
Peptide-based	Easily designed and synthesized Easily modified through carboxylic or amino groups for the incorporation of other supramolecular moieties Nanofibrous network formation resembles natural ECM structure Biodegradable Non-toxic Some peptides have intrinsic bioactivity	Weak mechanical properties and not adequate for certain TE applications pH related solubility Less stable Protein inactivation due to strong peptide-protein interactions

**Table 3:** Examples of model macromolecules released from supramolecular hydrogels through different release mechanisms.

Hydrogel Components	Hydrogel Type	Driving Force in Hydrogel Formation	Protein Loading Methods	Model Macromolecules	Release Period	Driving Force in Protein Release	Reference
HA-β-CD; HA-Ad	polymer	host-guest interaction	physical entrapment	BSA	60 days	erosion/ diffusion	[16]
HA-β-CD; HA-Azo	polymer	host-guest interaction	physical entrapment	BSA	8 days	stimuli (light)/ diffusion	[17]
PEG_8_- Cholesterol; PEG_8_-β-CD	polymer	hydrophobic and van der Waals interactions	physical entrapment	lysozyme; BSA	250 h	erosion/ diffusion	[23]
γ-CD; PCL-PEG-PCL	polymer	hydrophobic interactions	physical entrapment	insulin	37 days	diffusion/ erosion	[26]
α-CD; Py-PCL-b-POEGMA	polymer	host-guest interaction	physical entrapment	DOX; BSA	64 h	stimuli (temperature)	[33]
dex-HEMA-MAA; dex-HEMA-DMAEMA	polymer	electrostatic interactions	physical entrapment	IgG, BSA, lysozyme	60 days	diffusion	[68]
PVA-MV, HEC-Np, CB[8]	polymer	host-guest interaction	physical entrapment	BSA, lysozyme	20–160 days	diffusion	[69]
UPy-X-PEG-Zk (X = (CH_2_)_n_; Z = molecular weight of PEG)	polymer	hydrogen bonding	physical entrapment	CFP	4000 min	erosion	[70]
oleoylamide glycosyl-nucleoside-lipid	polymer (nucleoside-lipid)	hydrogen bonding; hydrophobic interactions; π-π stacking	physical entrapment	dextrans; IgG	-	stimuli (shear-mediated)	[71]
Ac-(RADA)_4_-NH_2_; Ac-(KLDL)_3_-NH_2_	peptide	electrostatic interactions	physical entrapment	IgG	100 days	diffusion	[40]
Ac-(RADA)_4_-NH_2_	peptide	electrostatic interactions	physical entrapment	IgG, BSA, lysozyme trypsin inhibitor	30–50 h	diffusion	[67]
MAX1/MAX8	peptide	electrostatic interactions	physical entrapment	dextran, lactoferrin	30 days	diffusion	[72]
MAX8	peptide	electrostatic interactions	physical entrapment	lysozyme, IgG, lactoferrin, α-lactalbumin, myoglobin, BSA	30 days	diffusion	[73]
HLT2 or VEQ3	peptide	electrostatic interactions	electrostatic interactions	α-lactalbumin, myoglobin, lactoferrin	4–28 days	diffusion	[74]
H-FEFQFK-NH_2_	peptide	hydrophobic interaction	physical entrapment	4-amino-2-cyclohexylmethyl-indolo[3,4-c]azepin-2-on (small molecule); 15 residue peptide; cAbVCAM1-5 Nb (protein)	12 h	diffusion (small molecule)/erosion (peptide, protein)	[75]
Ac-IKFQFHFD-NH_2_	peptide	π-π stacking; electrostatic interactions	physical entrapment (cypate); possible interactions with peptide due to structural similarities (proline)	cypate; proline	24 h; 75 h	stimuli (pH)	[76]
Ac-I_3_SLKG-NH_2_	peptide	hydrophobic interactions; hydrogen bonding	physical entrapment	peptide G3	16 days	stimuli (enzyme)	[77]
N4-octanoyl-2′-deoxycytidine	nucleoside	hydrogen bonding	non-covalent protein-gel association	β-lactoglobulin, BSA, lysozyme, insulin	24 h	erosion	[78]
PA/PSS	multi-component (peptide/ polymer)	electrostatic interactions	electrostatic interactions	BSA, lysozyme	30 days	diffusion	[61]
APmoc-F(CF_3_)F-OH/agarose	multi-component (peptide/ polymer)	hydrophobic interaction	physical entrapment	myoglobin	3 h	stimuli (enzyme)	[79]
HSA/DNA	multi-component (protein/ DNA)	multi-arm DNA crosslinking	ssDNA hybridization	GFP; YFP	30 min (trypsin) 15 min (DNase I)	enzyme (DNase I & trypsin) -mediated degradation	[80]
PAM/DNA	multi-component (polymer/ DNA)	DNA hybridization	thrombin-binding aptamer	human α-thrombin	-	reversible sol–gel transition by DNA strand displacement	[81]

Ac = acetyl group; Ad =adamantane; APmoc = acetoxybenzyl-oxycarbonyl group; Azo = azobenzene; BSA = bovine serum albumin; Bz = benzoate group; CB[8] = cucurbit[8]uril; CD = cyclodextrin; CFP = cyano-fluorescent protein; dex = dextran; DMAEMA = dimethylaminoethyl methacrylate; DOX = doxorubicin; GFP = green fluorescent proteins; HA = hyaluronic acid; HEC = Hydroxyethylcellulose; HEMA = 2-hydroxyethyl methacrylate; HSA = human serum albumin; IgG = immunoglobulin G; MAA = metha-crylic acid; MV = viologen; Np = naphthyl; PA = peptide amphiphile; PAM = polyacrylamide; PCL= polycaprolactone; PEG = poly(ethylene glycol); POEGMA = poly(oligo(ethylene glycol) methacrylate); PSS = poly(styrenesulfonate); PVA = poly(vinyl alcohol); Py = pyrene; Tyr = tyramine; UPy = ureidopyrimidinone; YFP = yellow fluorescent proteins.

**Table 4:** Therapeutic proteins delivered by supramolecular hydrogels for potential TE applications.

Therapeutic Protein(s)	Hydrogel	Release Period	Application	In Vivo Model	Reference
VEGF/FGF-2	PA-heparin	10 days	angiogenesis	rat cornea angiogenesis	[58]
VEGF_165_/TGF-β1/FGFβ	RAD16-I/heparin	36 h	angiogenesis	-	[88]
VEGF	SF/NapFF-RGD	21 days	angiogenesis	mice model	[89]
VEGF	RADA16/RADA16-PEG-PLGA	>30 days	angiogenesis	-	[104]
BMP-2	NapFFY nanofiber	35 days	bone regeneration	critical-sized periodontal bone defect models of maxillae in rats	[90]
BMP-2	BMP-2-binding PA nanofibers	28 days	bone regeneration	posterolateral lumbar intertransverse spinal fusion model in rats	[93]
BMP-2	Pluronic^®^127/ Tetronic^®^1307/α-CD	>7 weeks in vitro; 2 weeks in vivo	bone regeneration	osteoporosis model in rats	[105]
BMP-2	DEX-UPy	>1 month	bone-cartilage complex	subcutaneous implantation model in nude mice	[92]
TGF-β1	Ac-β-CDs/gelatin	>28 days	cartilage regeneration	knee osteochondral defects in rats	[94]
TGF-β1	Ac-β-CDs/HA-Ad	> 21 days	cartilage regeneration	knee osteochondral defects in rats	[96]
TGF-β3	monoCB[6]/DAH-HA	>3 weeks	cartilage regeneration	mice model	[97]
TGF-β1	TGF-β binding PA nanofibers	>72 h	cartilage regeneration	chondral defect microfracture model in rabbits	[106]
EGF	HA-β-CD/HA-Azo	depending on the provided light stimuli	skin healing	excisional full-thickness wound model in rats	[99]
bFGF	CS-Ag	>11 days	skin healing	infected wound model in mice	[100]
HGF/IGF-1	UPy-PEG	7 days	cardiac repair	porcine model of chronic ischemia	[34]
EPO	α-CD/MPEG-PCL-MPEG	>7 days	cardiac repair	myocardial infarction model in rats	[101]
anti-TGF-β/IL-10	HA-β-CD/HA-Ad	13 days	kidney	unilateral ureteral obstruction model in mice	[103]
BMP-7	UPy-X-PEG-Zk (X = (CH_2_)_n_; Z = molecular weight of PEG)	1 week	kidney	rats	[70]

Ac = acryl group; Ad = adamantane; Azo = azobenzene; BMP = Bone morphogenetic protein; CB[6] = cucurbit[6]uril; CD = cyclodextrin; CS = chitosan; DAH = diaminohexane; DEX = dextran; EGF = epidermal growth factor; EPO = erythropoietin; FGF = fibroblast growth factor; HA = hyaluronic acid; HGF = Hepatocyte growth factor; IGF = insulin-like growth factor; IL = interleukin; MPEG = methoxypolyethylene glycol; PA = peptide amphiphile; PCL = polycaprolactone; PEG = poly(ethylene glycol); PLGA = poly(lactic-co-glycolic acid); SF = silk fibroin; TGF = transforming growth factor; UPy = ureidopyrimidinone; VEGF = Vascular endothelial growth factor.

**Table 5:** Challenges in supramolecular hydrogels as protein delivery systems and proposed solutions.

Challenges	Solutions
Potential toxicity of the crosslinkers used (e.g., metals) or components are non-biodegradable or less biocompatible	Use nontoxic crosslinkers or at low concentrations Use biodegradable and biocompatible materials such as natural polymers or peptides
Burst release or less controllable protein release	Increase crosslink density Increase the interaction between proteins and hydrogel networks Use multicomponent hydrogels
Decrease in protein activity upon loading or release	Increase the stability of the hydrogel Reduce strong interactions between proteins and hydrogel networks Use protein-friendly crosslinking chemistries
Inappropriate mechanical properties	Use multicomponent hydrogels
Slow sol-gel transition after injection	Increase the crosslinking density Increase the interaction affinity between hydrogel components Use materials responsive to local stimuli
Slow gel-sol transition	Increase the intensity of applied stimuli if they are external Incorporate additional reversible crosslinks sensitive to stimuli
